# Analyzing the effect of sleep duration, chronotype, and social jet lag on anxiety disorders and health-related quality of life: A cross-sectional study

**DOI:** 10.1371/journal.pone.0314187

**Published:** 2024-11-21

**Authors:** Sung Min Jung, Mee-Ri Lee

**Affiliations:** 1 Department of Surgery, Inje University, Ilsan Paik Hospital, Goyang, Republic of Korea; 2 Department of Preventive Medicine, Soonchunhyang University College of Medicine, Cheonan, Republic of Korea; Federal University of Rio Grande do Sul: Universidade Federal do Rio Grande do Sul, BRAZIL

## Abstract

**Background:**

Anxiety disorders significantly impair the quality of life (QOL). Studies on the effect of sleep duration, chronotype, and social jet lag on anxiety disorders are limited. This study aimed to elucidate the contributions of sleep duration, chronotype, and social jet lag to the prevalence and severity of anxiety disorders in Koreans.

**Methods:**

This study used data of 9,874 Korean adults from the Korea National Health and Nutrition Examination Survey 2021–2022. Anxiety was assessed using the Generalized Anxiety Disorder-7 scale, and health-related QOL was measured using the Health-Related Quality of Life Instrument with 8-Items. Complex sample logistic regression models were used to evaluate the associations between sleep duration, chronotype, and social jet lag and anxiety disorders, with analyses stratified by sex.

**Results:**

Sleep duration of <6 h, evening chronotype, and social jet lag of >2 h were significantly associated with a high prevalence of anxiety disorders in women; however, in men, only sleep duration of <6 h was associated with anxiety disorders. Sleep duration of <6 h and evening chronotype were associated with low QOL in women but not in men.

**Conclusion:**

This study highlights the importance of adequate sleep and circadian alignment for mental health, particularly in women. Public health initiatives should focus on promoting healthy sleep habits to improve mental health and QOL. Further research is required to understand the causal pathways and sex-specific mechanisms underlying these associations.

## Introduction

Anxiety disorders are one of the most prevalent mental health conditions. Approximately 301 million people worldwide have been estimated to experience anxiety disorders [1]. Anxiety disorders such as generalized anxiety disorder (GAD), social anxiety disorder, and panic disorder affect 6.2%, 13%, and 5.2% of the population, respectively, and manifest as worry, social and performance anxiety, panic attacks, and avoidance behaviors [2]. Anxiety disorders markedly impair health-related quality of life (HRQoL) and have a bidirectional effect on mental health [3]. Individuals diagnosed with GAD face an elevated risk of suicide and a high likelihood of cardiovascular events and mortality [4]. Moreover, older adults are at an increased risk of cognitive decline [5]. In primary care settings, brief screening tools, such as the Generalized Anxiety Disorder-7 (GAD-7), are instrumental in diagnosing anxiety disorders, demonstrating a sensitivity of 57.6–93.9% and specificity of 61–97% [2].

While the impact of circadian rhythm on mental health has been recognized [6], studies exploring the relationship of chronotype, social jet lag, and sleep duration with anxiety disorders and HRQoL remain scarce. A recent Mendelian randomization study showed that short or long sleep duration was not associated with anxiety in the Chinese population [7]. In older Japanese women, HRQoL scores showed no significant differences among groups with varying sleep midpoints [8].

Using data from the Korea National Health and Nutrition Examination Survey (KNHANES), this study aimed to elucidate the contributions of sleep duration, chronotype, and social jet lag to the prevalence and severity of anxiety disorders in Koreans.

## Methods

### Study design

**Fig 1:**
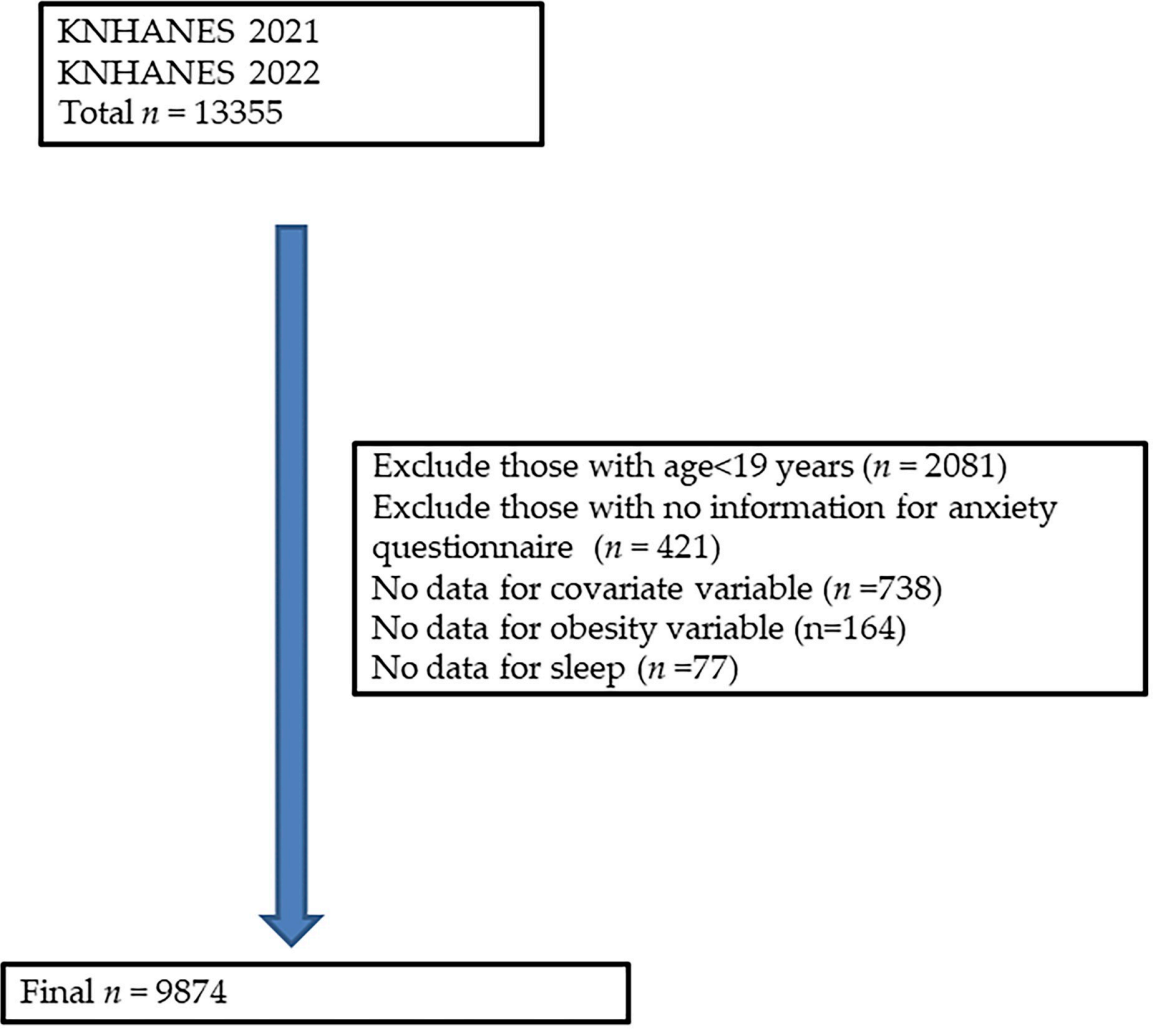
Flow chart for study population.

This nationally representative cross-sectional study used data from the KNHANES 2021–2022 which is a national dataset, conducted by the Korea Centers for Disease Control and Prevention. In accordance with Korean standards, individuals aged ≥19 years were considered adults. The final sample comprised 9,874 participants, after excluding minors (those under 19 years of age) and individuals lacking data on anxiety assessments, sleep variables, obesity variables, and covariates of interest (Fig 1).

### Ethics statement

The KNHANES studies were conducted in accordance with the principles of the Declaration of Helsinki. Informed consent was obtained from all subjects involved in the KNHANES study. Participants in this study were extracted from publicly available big data that had been de-identified, making it impossible to identify the subjects or obtain consent forms. This study was approved by the Institutional Review Board of Inje University Ilsan Paik Hospital (ISPAIK 2024-02-012).

### Generalized Anxiety Disorder-7

In our investigation, the GAD-7 scale, presented in its Korean version, was employed to quantify anxiety levels among participants. This self-report measure assesses the frequency of anxiety symptoms over the previous 2 weeks, with items rated on a scale ranging from 0 (not at all) to 3 (nearly every day), yielding a total score range of 0 to 21. The total GAD-7 score categorizes anxiety severity into four levels: normal (0–4 points), mild (5–9 points), moderate (10–14 points), and severe (15–21 points). For the purposes of this study, a score of ≥10 was established as the cutoff point to identify cases of moderate-to-severe anxiety, aligning with the instrument’s validation research, which confirmed its reliability and validity in both clinical and general populations. Approximately 5% of individuals in a normative sample scored ≥10, underscoring the relevance of this threshold for anxiety assessment [9].

### Health-Related Quality of Life

The Health-Related Quality of Life Instrument with 8 Items (HINT-8) was used to assess HRQoL. The HINT-8 consists of eight items and four responses ranging from 0.132 (worst) to 1 (best). The details of the HINT-8 have been described in a previous article and on the KNHANES website [10]. To analyze each item of the HINT-8, we divided the answers into 2 groups: “no” was designated to the “no problem” group and “mild,” “moderate,” and “severe” were designated to the “having problem” group. The HINT-8 was administered only in 2021. Those with HINT-8 scores of <10% were defined as having a low HRQoL.

### Sleep

#### Sleep duration

Sleep-related data were collected differently over the 2 years. In 2021, the survey measured the number of times participants fell asleep and were awake on weekdays and weekends. The questionnaire asked, ’What time do you usually go to bed and wake up during the week (or on workdays)?’ and ’What time do you usually go to bed and wake up on weekends (or on days off, or the night before a day off)?’ The average daily sleep duration on both weekdays and weekends was assessed for the year 2022. The sleep duration in 2021 was calculated using the difference between wake time and bedtime, whereas in 2022, the sleep duration was determined by averaging the sleep time across the week ([weekday sleep time × 5 + weekend sleep time × 2] / 7). In line with previous studies, we categorized sleep duration as follows: short (<6 h of sleep per day), normal (6–8 h of sleep per day), and long (≥9 h of sleep per day) [11].

#### Chronotype

We calculated the mid-sleep time on free days (MSF) as the sum of the sleep onset time during the weekends and half of the total sleep duration on these days. The sleep-corrected mid-sleep time on free days (MSFsc) was then derived by adjusting the MSF for accumulated sleep debt across the week, using the following formula: MSFsc=MSF−0.5×(weekendtotalsleeptime−[5×weekdaytotalsleeptime+2×weekendsleeptime]/7) [12]. This approach provided a refined metric for assessing circadian rhythm adjustments relative to sleep debt. Based on MSFsc, chronotype classification was divided into the following three categories: morning chronotype, defined as an MSFsc less than the mean minus one standard deviation (SD); intermediate chronotype, defined as an MSFsc between the mean minus one SD and the mean plus one SD; and evening chronotype, defined as an MSFsc greater than the mean plus one SD [13]. The assumption was that all participants had 5 workdays and 2 free days per week. The use of an alarm clock was not factored into the classification.

#### Social jet lag

Previous research has identified social jet lag as a discrepancy in sleep timing between free days and workdays, specifically comparing the midpoint of sleep on these days (MSF vs. mid-sleep time on workdays) [14]. Similar to MSFsc, the sleep-corrected midpoint of sleep on workdays (MSWsc) was estimated by adding half of the average sleep duration to the sleep onset time on workdays [15]. Consequently, the sleep-corrected social jet lag (SJLsc) was calculated as the absolute difference between MSFsc and MSWsc, using the following formula: “SJLsc = |MSFsc–MSWsc|” [16]. Social jet lag was defined as a discrepancy of >2 h differences in sleep and wake times across weekdays and weekends [17]. This approach allowed for the assessment of chronotypes and social jet lag using the 2021 data, for which sleep onset and wake times were available.

### Covariates

The participants’ educational level, smoking habits, alcohol consumption, and physical activity were evaluated using self-administered questionnaires. Education was categorized into three levels: low (<9 years), medium (9–11 years), and high (≥12 years). Smoking status was classified into three groups: lifetime nonsmokers, former smokers (individuals who had smoked in the past but had ceased smoking at the time of the study), and current smokers (individuals who had smoked at least 100 cigarettes in their lifetime and continued to smoke). Alcohol consumption was assessed by identifying participants who reported consuming one or more alcoholic drinks per month in the past year. Physical activity is defined as 150 minutes or more of moderate-intensity physical activity per week, 75 minutes or more of high-intensity physical activity per week, or a combination of moderate- and high-intensity physical activity (classified according to the time spent on each activity) [18]. Body mass index (BMI) was calculated as weight divided by height squared (kg/m^2^). Overweight was defined as having a BMI of ≥25 kg/m^2^.

### Statistical analyses

Data from the KNHANES for the years 2021–2022 were merged for analysis. To accommodate the multilevel sampling design of the study, complex sample analysis techniques were employed, utilizing sampling weights in all analyses to ensure the representativeness of the national population. The association between sleep patterns and risk of developing anxiety disorders was evaluated using logistic regression models. These models generated odds ratios (OR) and 95% confidence intervals (CI) as measures of association.

Given the presence of sex-based differences, all analyses were conducted for all participants and separately for men and women.

To account for potential confounders, multivariable models were adjusted for several known risk factors for anxiety disorders. These included age (as a continuous variable), education level (categorized as low, medium, high), smoking status (never smoker, former smoker, or current smoker), alcohol consumption (non-binge drinker or binge drinker), physical activity level (inactive or active), and BMI. Statistical significance was determined using a p-value of 0.05.

Statistical analyses and graphical presentations were conducted using Stata, version 17 (Stata Corp., College Station, TX, USA), and R software, version 4.0.2 (R Core Team, Vienna, Austria), respectively.

## Results

This study analyzed data of 9,874 participants, comprising 4,339 men and 5,535 women, from KNHANES 2021–2022. Anxiety disorders, measured using the GAD-7 scale, showed a prevalence of anxiety (GAD-7 score ≥10) in 138 men (3.2%) and 331 women (6.0%). The proportions of individuals with sleep durations of <6, 6–8, and ≥9 h were 13.2%, 78.9%, and 7.9%, respectively. The proportions of individuals with morning, intermediate, and evening chronotypes were 15.6%, 71.1%, and 13.4%, respectively, and the proportion of those with >2 h of SJLsc was 2.34% in 2021 data.

Statistically significant differences were observed in terms of age, with younger participants being more likely to report anxiety disorders than older participants (Table 1). The smoking status and alcohol consumption patterns varied, with women showing significant differences in alcohol consumption between those with and without anxiety (Table 1). Education level, physical activity, and BMI (≥25 kg/m^2^) were not significantly correlated with anxiety (Table 1). Sleep duration was significantly different in participants with anxiety in the total population (p<0.001), and evening chronotype and social jet lag of >2 h were prevalent among those with anxiety, particularly in women (p<0.001 and p = 0.001, respectively) (Table 1).

**Table 1:** Participants’ sociodemographic and clinical characteristics according to anxiety disorder and sex.

Variable	Men (n = 4339)			Women (n = 5535)			Total (n = 9874)		
KNHANES 2021, 2022	GAD-7<10	GAD-7≥10	*P* value	GAD-7<10	GAD-7≥10	*P* value	GAD-7<10	GAD-7≥10	*P* value
n	4201	138		5204	331		9405	469	
Age, y,	52.9±17.3	48.7±17.4	**0.006**	53.2±16.7	49.6±18.4	**<0.001**	53.0±16.9	49.4±18.1	**<0.001**
Education									
<9	827(12.1)	24(8.8)	0.369	1557(21.9)	100(20.2)	0.801	2384(16.9)	124(16.3)	0.916
9–11	1194(27.8)	37(25.7)		1395(27.8)	85(28.2)		2589(27.8)	122(27.4)	
≥12	2180(60.2)	77(65.6)		2252(50.3)	146(51.6)		4432(55.3)	223(56.3)	
Smoking status									
Never smoker	1053(26.8)	31(25.3)	0.691	4635(88.3)	254(74.4)	**<0.001**	5688(57.0)	285(57.9)	0.247
Former smoker	1943(43.4)	56(41.1)		342(6.9)	37(12.6)		2285(25.5)	93(22.1)	
Current smoker	1205(29.8)	51(33.6)		227(4.8)	40(13.0)		1432(17.5)	91(20.0)	
Alcohol drinker	2726(66.9)	86(68.2)	0.772	1963(40.8)	150(50.2)	**0.010**	4689 (54.1)	236(56.2)	0.462
Physical activity	1948(50.4)	65(54.1)	0.423	2154(44.6)	146(47.7)	0.357	4102(47.6)	211(49.8)	0.392
BMI≥25 (kg/m^2^)	1814(45.9)	56(43.8)	0.653	1582(28.2)	122(34.0)	0.063	3396(37.2)	181(37.3)	0.9715
Sleep duration									
Short (<6)	493(11.0)	27(16.8)	0.115	707(12.8)	78(21.0)	**<0.001**	1200(11.9)	105(19.6)	**<0.001**
Normal (6–8)	3386(82.4)	100(76.6)		4085(79.9)	218(70.1)		7471(81.1)	318(72.3)	
Long (9≤)	322(6.6)	11(6.6)		412(7.4)	35(8.9)		734(7.0)	46(8.1)	
**KNHANES 2021**									
n	2206	71		2686	161		4892	232	
Chronotype									
Morning	385(12.0)	11(9.5)	0.480	378(9.9)	23(9.6)	**<0.001**	763(11.0)	34(9.6)	**0.002**
intermediate	1513(70.0)	46(46.0)		1983(74.2)	101(62.3)		3496(72.1)	147(63.8)	
Evening	308(18.0)	14(23.7)		325(15.9)	37(28.2)		633(17.0)	51(26.6)	
Social jetlag>2 hours	66(3.2)	3(4.7)	0.561	42(2.0)	9(8.5)	**0.001**	108(2.6)	12(7.22)	**0.008**
Low Hint-9	108(3.5)	30(34.0)	**<0.001**	283(8.5)	92(51.0)	**<0.001**	391(5.9)	122(45.3)	**<0.001**

Data were presented as number (percentage) or mean ± standard deviation. χ^2^ test and t-test were used for categorical and continuous variables, respectively.

Bold numbers highlight the statistical significance.

Abbreviations: BMI, body mass index; GAD-7, Generalized Anxiety Disorder-7; KNHANES, Korea National Health and Nutrition Examination Survey.

**Fig 2:**
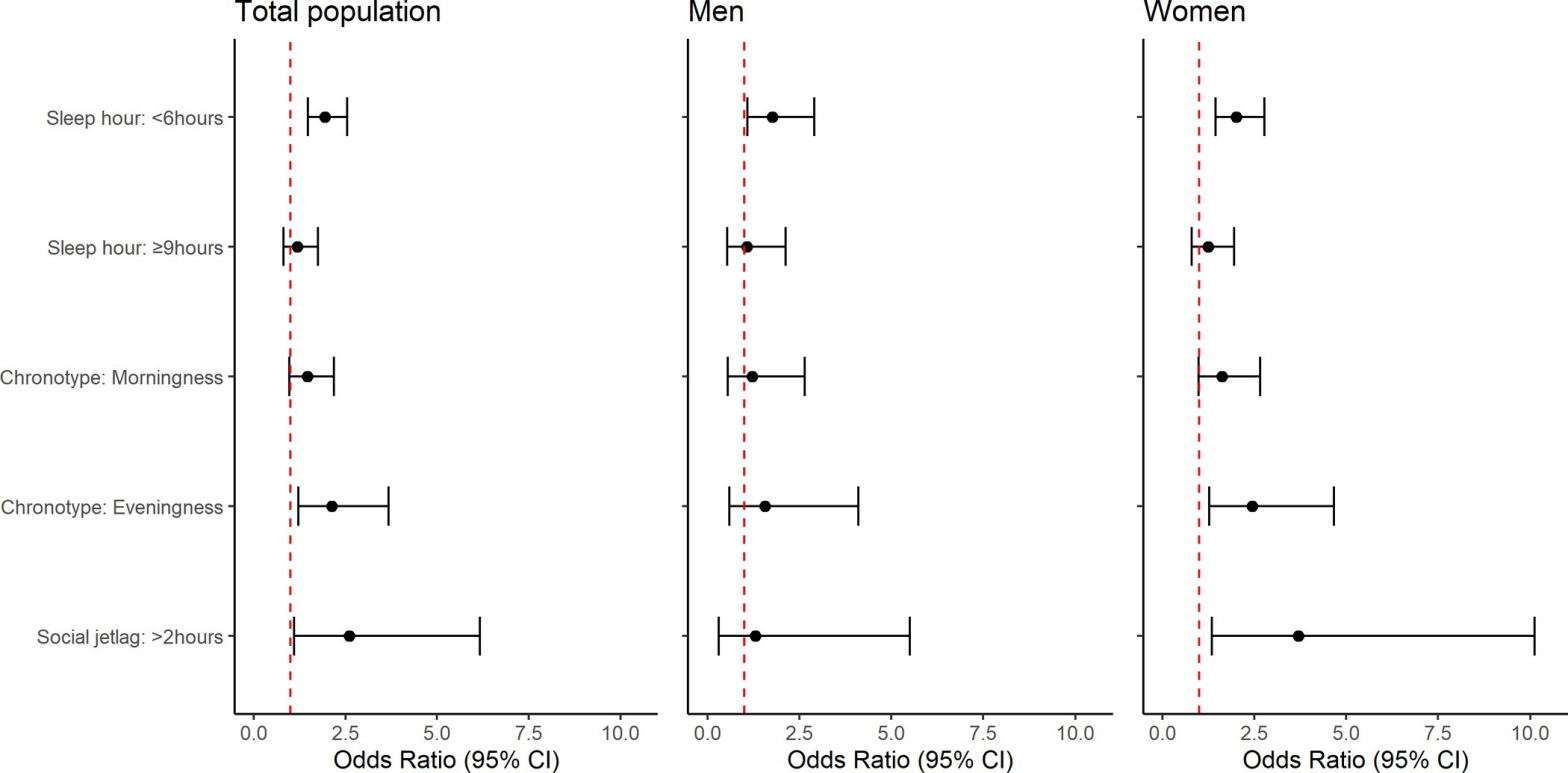
Sleep factors related with anxiety disorder using complex sample logistic regression by sex.

Fig 2 presents the outcomes of a complex sample logistic regression examining the association between sleep factors and anxiety disorders across the sexes. For the total population, sleep duration of <6 h (OR: 1.94, 95% CI: 1.47–2.55), evening chronotype (OR: 1.58, 95% CI: 1.02–2.46), and social jet lag of >2 h (OR: 2.61, 95% CI: 1.10–6.17) were identified as potential risk factors associated with anxiety disorder. When disaggregated by sex, a similar trend in correlation of sleep duration of <6 h, evening chronotype, and social jet lag with anxiety disorder (OR: 2.00, 95% CI: 1.44–2.77; OR: 1.75, 95% CI: 1.00–3.04; OR: 3.70, 95% CI: 1.35–10.12, respectively) was observed in women. However, in men, no significant associations between sleep factors and anxiety disorder, except for sleep duration of <6 h (OR: 1.77, 95% CI: 1.08–2.90), were observed.

**Fig 3:**
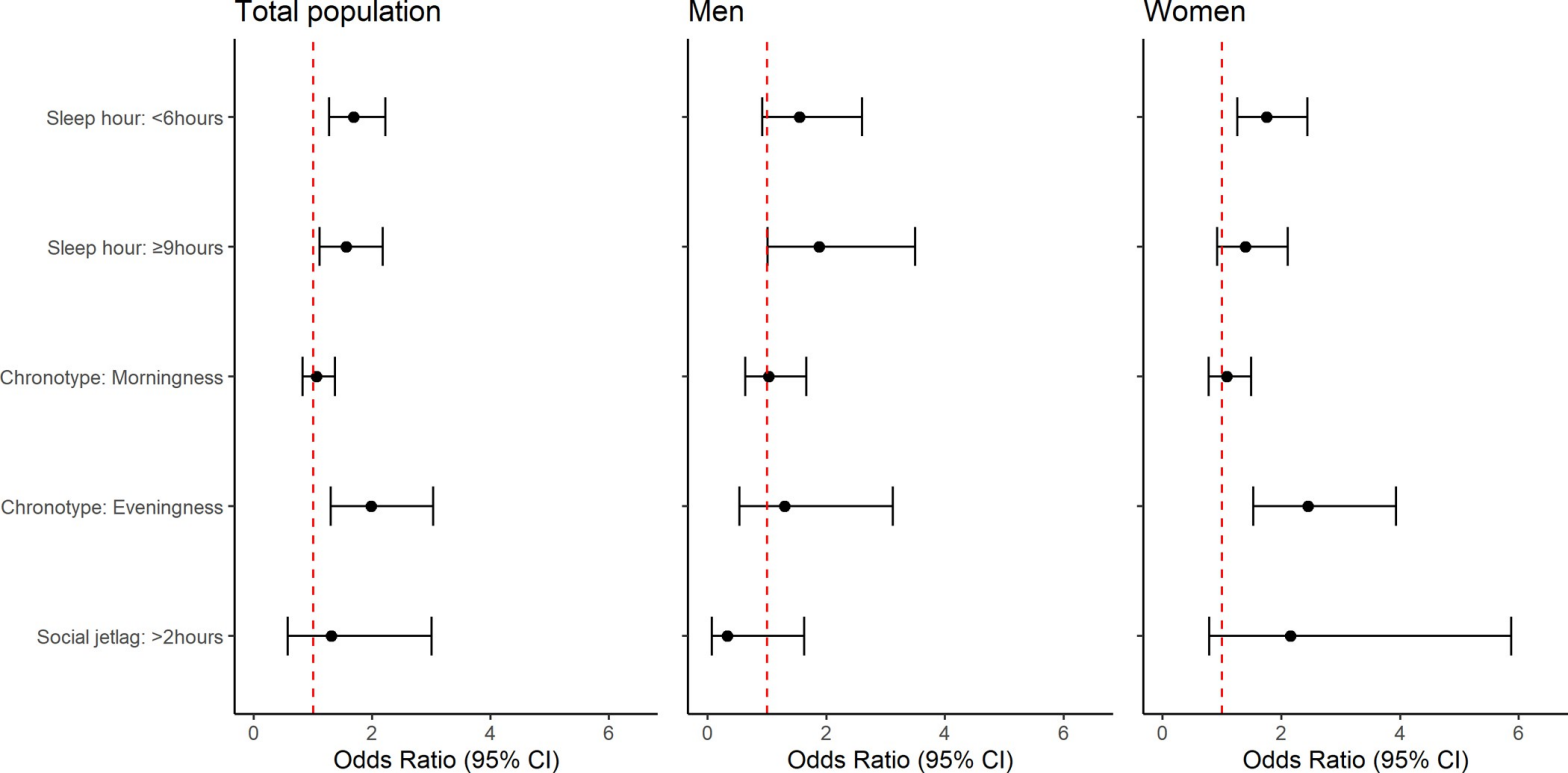
Sleep factors related with quality of life using complex sample logistic regression by sex.

Fig 3 illustrates the outcomes of a complex sample logistic regression exploring the associations between sleep factors and quality of life, differentiated by sex. For the overall population, the analysis identified several risk factors associated with the quality of life: <6 h of sleep per night (OR: 1.68, 95% CI: 1.27–2.22), >9 h of sleep per night (OR: 1.56, 95% CI: 1.11–2.18), and evening chronotype (OR: 1.98, 95% CI: 1.30–3.03). In women, factors such as <6 h of sleep (OR: 1.75, 95% CI: 1.26–2.44) and evening chronotype (OR: 2.45, 95% CI: 1.53–3.93) were significantly associated with low quality of life. Conversely, in men, none of these factors showed significant associations with quality of life, except for >9 h of sleep per night (OR:1.88, 95% CI: 1.01–3.50).

## Discussion

This study used data of 9,874 participants from the KNHANES for the years 2021–2022 to explore the relationship between sleep factors and mental health, particularly focusing on anxiety disorders and HRQoL in different sexes By establishing this link, our findings contribute to the growing body of evidence that highlights the impact of circadian rhythm disruptions, specifically anxiety disorders, on mental health.

The lifetime prevalence of GAD exhibits significant global variation, with rates ranging from <1% in Nigeria and Shenzhen (China) to approximately 8% in Australia, New Zealand, and the United States, positively correlating with economic development [19]. Demographically, GAD is more prevalent among females and individuals <60 years [19]. Similarly, in our study, anxiety disorders were measured using the GAD-7 scale with a cutoff value of 10, revealing a prevalence of 3.2% in men and 6.0% in women, with higher rates observed in females and individuals aged <60 years than in those aged ≥60 years (5.27% vs. 3.98%).

The distribution of sleep duration was primarily in the 6–8 hour range, with a smaller percentage reporting sleep durations of <6 hours or >9 hours. Notably, sleeping for <6 hours was statistically associated with anxiety in the total population (p<0.001), underscoring the potential role of insufficient sleep in anxiety disorders. Additionally, evening chronotype and significant social jet lag (>2 hours) were more common among individuals with anxiety, particularly among women, suggesting that misalignment of natural sleep patterns may be linked to anxiety. Our findings are consistent with those of previous studies. Data of 2,619 Dutch showed that short sleep hours (<6 h) were associated with current anxiety disorder (OR: 1.41, 95% CI: 1.13–1.77) compared with never anxiety disorder [20]. Zhou et al. [21] showed that the combined effect of short sleep hours (<7 h) and late sleep initiation time (≥24:00) had the greatest odds (OR, 2.82) of prevalent anxiety symptoms in 28,054 Chinese rural adults. Merikanto and Partonen [22] found that among 18,039 Finnish adults, those with an evening chronotype exhibited increased anxiety symptoms, a relationship partly mediated by insufficient sleep. In a cross-sectional epidemiological study across three inpatient clinical settings, 1,468 consecutive inpatients with anxiety disorders were likely to have an evening chronotype [23]. A study showed that evening chronotypes significantly increased anxiety and poor general well-being of 136 Australian paramedics [24].

Our study also explored the associations between sleep factors and HRQoL, revealing that insufficient sleep (<6 h), excessive sleep (>9 h), and evening chronotype were correlated with lower quality of life in the overall population. These findings are consistent with previous research, suggesting that both sleep duration and circadian preferences may have a significant influence on well-being. A recent study showed that among Chinese college students, those who slept for ≥9 h per day had a significantly higher mental component summary score of HRQoL than those who slept for 7–8 h per day [25]. In a Korean study using multiple linear regression, both short and long sleep durations were associated with lower EQ-5D index and EQ-VAS scores than those of 7-h sleepers [26]. Recent findings indicate that both short and long sleep durations were significantly negatively correlated with HRQoL utility scores in older adults in the United Kingdom [27].

Insufficient sleep is associated with increased activity of the sympathetic nervous system, hypothalamic-pituitary-adrenal axis, and inflammatory responses, resulting in reduced quality of life and mood disorders [28]. Individuals with evening chronotype and social jet lag experience circadian misalignment between their internal clock and societal demands, leading to chronic sleep debt, stress, and fluctuations in melatonin and cortisol hormones, thereby impacting mood and anxiety levels [29, 30].

Our study found that both men and women showed an association between short sleep durations and anxiety. Additionally, in women, evening chronotype and social jet lag were significantly associated with anxiety. In terms of HRQoL, long sleep duration was associated with lower quality of life in men, while short sleep duration and evening chronotype were associated with lower quality of life in women. Women have hormonal fluctuations across the menstrual cycle, and estradiol can heighten the hypothalamic-pituitary-adrenal axis response to stress, potentially leading to increased anxiety and fatigue and indirectly affecting sleep [31]. Women also have higher psychosocial distress and caregiving responsibilities, higher strain associated with short sleep and evening chronotypes, and worse mental health than men [32]. In a study, women had more N3 sleep (the most restorative sleep stage) and were less awake after sleep onset than men, after adjusting for age [33]. Disruptions in these patterns owing to evening chronotype or social jet lag may have a pronounced effect on mental health. Some studies suggest that women may be more biologically vulnerable to sleep disturbances [34] and anxiety disorders than men because of the inflexibility of stimulus-response associations and behavioral inhibition [35]. Korean women often have low social status and face labor precarity, resulting in relatively poor mental health [36].

This study had several limitations. First, the cross-sectional nature of the study limited our ability to establish causal relationships. Longitudinal studies are required to confirm the directionality of the observed associations. Second, only Korean adults were included; therefore, the findings cannot be generalized to children, adolescents, or populations of other countries. Third, self-reported measures of sleep duration and anxiety may have been subject to recall bias. Objective measures, such as actigraphy for sleep and clinical assessment of anxiety, would strengthen future research. Finally, a validated questionnaire was not used to assess social jet lag, and the validity of the core questions used has not been confirmed.

## Conclusion

Our study highlights notable sex differences in the relationship of sleep-related factors with anxiety disorders and HRQoL. Specifically, a short sleep duration significantly associated with anxiety in both men and women. However, in women, evening chronotype and social jet lag were associated with anxiety. In terms of HRQoL, long sleep duration was a crucial factor in men, whereas short sleep duration and evening chronotypes played significant roles in women. These findings underscore the importance of considering sex-specific sleep patterns and chronotypes when assessing and managing anxiety and HRQoL. Further research is warranted to explore tailored interventions that can effectively address these differences.
